# First steps in designing an all-in-one ICT-based device for persons with cognitive impairment: evaluation of the first mock-up

**DOI:** 10.1186/s12877-016-0238-x

**Published:** 2016-03-07

**Authors:** Inga-Lill Boman, Ann-Christine Persson, Aniko Bartfai

**Affiliations:** University Department of Rehabilitation Medicine, Danderyd Hospital, S-182 88 Stockholm, Sweden; Department of Clinical Sciences, Karolinska Institutet, Danderyd Hospital, S-182 88 Stockholm, Sweden

**Keywords:** Aging, Ambient assisted living, Assistive technology, Informal caregivers, Services, Quality of life

## Abstract

**Background:**

This project Smart Assisted Living involving Informal careGivers++ (SALIG) intends to develop an ICT-based device for persons with cognitive impairment combined with remote support possibilities for significant others and formal caregivers. This paper presents the identification of the target groups’ needs and requirements of such device and the evaluation of the first mock-up, demonstrated in a tablet.

**Methods:**

The inclusive design method that includes end-users in the design process was chosen. First, a scoping review was conducted in order to examine the target group’s need of an ICT-based device, and to gather recommendations regarding its design and functionalities. In order to capture the users’ requirements of the design and functionalities of the device three targeted focus groups were conducted. Based on the findings from the publications and the focus groups a user requirement specification was developed. After that a design concept and a first mock-up was developed in an iterative process. The mock-up was evaluated through interviews with persons with cognitive impairment, health care professionals and significant others. Data were analysed using content analysis.

**Results:**

Several useful recommendations of the design and functionalities of the SALIG device for persons with cognitive impairment were identified. The main benefit of the mock-up was that it was a single device with a set of functionalities installed on a tablet and designed for persons with cognitive impairment. An additional benefit was that it could be used remotely by significant others and formal caregivers.

**Conclusion:**

The SALIG device has the potentials to facilitate everyday life for persons with cognitive impairment, their significant others and the work situation for formal caregivers. The results may provide guidance in the development of different types of technologies for the target population and for people with diverse disabilities. Further work will focus on developing a prototype to be empirically tested by persons with cognitive impairment, their significant others and formal caregivers.

## Background

Persons with cognitive impairment might need several devices for support in everyday activities [1]. They might need time-cued prospective memory devices that can support them in remembering to perform activities at specific times [2]. Information and communication technology (ICT) such as computers, mobile phones, pagers and voice organisers have been used for a long time as prospective memory devices [3–5]. More recently, smartphones and tablets have been used to compensate for cognitive impairment [5, 6]. Smart technology has several functions, high storage capacity, and rich multimedia capability. An additional advantage is that the general population uses smart technology, since assistive technologies could be perceived as stigmatising [2, 7, 8]. However, a problem with smart technology is that persons with cognitive impairment might have difficulties in using it effectively [5, 9]. Another problem might be the small size and small buttons if the user has visual impairment or reduced fine motor skills [10, 11]. Consequently, tablets with lager screen may be easier to use. A dilemma is that due to the fast technical development taking place, there is a lack of research concerning the usability of tablets for persons with cognitive impairment [12].

Nevertheless, time-cued prospective memory devices might not be enough. Persons with cognitive impairment might also need event-cued devices [13] such as smart home technology that could monitor equipment and activities in the home environment. Smart home technology can monitor and send event-cued reminders if, for example, the user has forgotten to turn off the cooker or the tap, or close windows or the front door or to have meals [14]. Moreover, the installation of fall detection systems could also be important to increase security in the home. Persons with cognitive impairment might also need telepresence technology such as the robot Giraff (www.giraff.org) for guidance and support in everyday activities and to facilitate communication [15]. Easy-to-use video communication solutions might also be useful to maintain social interaction [16].

Even if smart technology could be useful for persons with cognitive impairment it might be difficult for them to learn how to use and maintain several devices [17]. Persons with cognitive impairment might need support from significant others (SOs) and formal caregivers (FCs) to be able to use several devices [18, 19]. A problem is that SOs often bear a huge burden in supporting persons with cognitive impairment in their everyday life and there is a need to lessen this burden [20]. There is a need to develop a single device with a set of functionalities that is easy-to use and could be shared with SOs in order to increase independence for persons with cognitive impairment and lessen the burden of their SOs. Today, there is a lack of such device that could be individually adapted to users’ needs and requirements. Furthermore, there is also a lack of knowledge concerning how to develop and match ICT solutions to meet the needs and requirements of persons with cognitive impairment [21, 22].

This project Smart Assisted Living involving Informal careGivers++, SALIG (www.salig.eu) intends to develop an all-in-one device that is based on ICT for persons with cognitive impairment after acquired brain injury and persons with mild and moderate dementia. The SALIG device will be able to monitor and respond to the users’ needs of support to maintain an independent life despite their cognitive impairment combined with possibilities for remote support for SOs and FCs. This paper reports the first steps in designing the SALIG device.

## Methods

There are many aspects to consider when developing a product for persons with cognitive impairment [16]. It has been suggested that the development of technology for persons with cognitive impairment requires a holistic person-centered approach with users involved in order to develop useful and easy-to-use products [23]. In this project we have used a modified Inclusive Design method that includes the target users in the design process [24]. The method comprises four phases: (1) examine the need of the user, (2) develop a user requirement specification of the design, (3) create a design concept, and (4) develop and evaluate a prototype and define a detailed plan for the final product.

### Ethics

The study was approved by the Regional Ethical Review Board in Stockholm, Karolinska Institutet, receiving journal number 2013/5:9. The participants received oral and written information about the study and that participation in the evaluation was voluntary. They all gave their written informed consent.

### Design process

#### Phase 1: examine the need of users

In the first phase the need of an ICT-based device for persons with cognitive impairment as support in managing everyday activities was examined through a scoping review [25]. Publications were searched in Amed, Cinahl, OT-seeker, PsycINFO and PubMed databases and on the Internet using Google. Moreover, we continuously retrieved other publications based on their references. The collected publications were reviewed in order to identify persons with cognitive impairment need of an ICT-based device as support in managing everyday activities. The data were summarised, coded and then categorised by using qualitative content analysis principles [26]. The collected data led to a description of the target users’ need of support in managing everyday activities (Table 1).

**Table 1:** Persons with cognitive impairment need of an ICT-based device as support in managing everyday activities

Domain	Needs of support
Personal Care	Remember to have a meal
	Remember to change clothes
	Remember to have a shower
Instrumental activities	Remember activities/appointments
of daily living	Initiate and complete activities
	Plan the week
	Remember to bring things (mobile, keys, wallet etc.)
	Remember to charge technology
	Remember to buy things
	Remember to take care of the laundry
	Remember to take out the rubbish
	Remember verbal information/series of instructions
	Remember names, numbers, user names, passwords
	Find lost items
Health/Well-being	Remember to take medication
	Remember to check blood sugar, blood pressure
	Remember to have a rest in the afternoon
	Remember to exercise
Safety/security	Remember to turn off home equipment, close windows
	Remember to lock the door
	If falling
	If getting lost

#### Phase 2. Develop a user requirement specification

In the next phase, requirements of the functionalities and design on the ICT-based device for persons with cognitive impairment were specified by using the same method of literature review as described above. The collected publications were reviewed in order to identify recommendations regarding the design and functionalities of an ICT-based device for persons with cognitive impairment. These data were analysed in the same way as described in phase 1. This led to an identification of a suggested design and functionalities of an ICT-based device for persons with cognitive impairment.

Even though the literature provided recommendations of design and functionalities we did not know what persons with cognitive impairment require. In order to capture the users requirements of the design and functionalities of the SALIG device, three targeted focus groups were formed. For a description of the participants’ characteristics see Table 2. We first turned to occupational therapists (OTs) working with persons with cognitive impairment as they have valuable clinical knowledge in this subject and have a central role in prescribing assistive technology (AT) [27]. OTs were recruited through a network of OTs working in this area. The OTs were required to have more than 2 more years’ experience in this position. Thereafter, we invited persons with cognitive impairment to the second focus group. Persons with cognitive impairment were recruited through OTs working in this area. Inclusion criteria for persons with cognitive impairment were that they had to be able answer open-ended questions, had a reasonable capacity to remember daily events and relate them to their cognitive problems and had a need for increased independence through use of ATs. Exclusion criteria were other concurrent somatic or psychiatric disease influencing participation. The third focus group consisted of SOs of persons with cognitive impairment. SOs were recruited through OTs and physiotherapists working in this area. SOs had to be a spouse, relative or close friend significantly familiar with the everyday life of the participating person with cognitive impairment. Potential participants were invited and informed by telephone and in writing. These three focus groups provided data considered rich enough to formulate a user requirement specification.

**Table 2:** Demographics of the participants in the three focus groups (occupational therapists, persons with cognitive impairment and significant others of persons with cognitive impairment)

Focus group 1: Occupational therapists (*n* = 6)	
Sex, male/female	0/6
Age, median (range), years	47 (40–54)
Professional experience, median (range) years	20 (14–29)
Focus group 2: Persons with cognitive impairment (*n* = 4)	
Sex, male/female	2/2
Age, median (range), years	58.5 (33–62)
Stroke (n)	4
Time since diagnosis, median (range), months	5.5 (5–36)
Memory deficits (n)	4
Focus group 3: Significant others of persons with cognitive impairment, PWCI (*n* = 4)	
Sex, male/female	2/2
Age, median (range), years	58 (52–68)
Relationship to PWCI	
Spouse (n)	2
Daughter	1
Son	1
Time since PWCI diagnosis, median, (range) years	5.25 (2–10)

The focus groups consisted of 4–6 participants; in total 14 persons. These rather small groups were chosen to facilitate elaboration of views and issues between participants as recommended in the literature [28]. The interviews with the focus groups were conducted in a hospital training apartment. Each session lasted between 1 and 2 h including refreshment breaks. The focus groups met for one session and the sessions were audio taped. The first author moderated the focus groups discussions with assistance from the second author. An interview guide with open questions was developed for the focus groups, covering ideas on design and functionalities of an ICT-based device. After each focus group session the first and the second author reflected on the discussion, also embracing immediate suggestions for issues that should be brought up in the next focus group. This meant that the interview guide was modified between the groups according to the principles of grounded theory [29] in order to cover additional relevant issues.

The collected data was analysed using principles of qualitative content analysis [26]. First, the digitally recorded focus group discussions were transcribed. After that, the material was read through in order to identify persons with cognitive recommendations of functionalities and design of an ICT-based device. In the next step, the collected data were summarised, coded and categorised. As the analysis proceeded the categories from the focus groups were compared with the categories from the scoping review and thereafter the categories were merged together. To be sure that the analysis was grounded in the data, the categories were constantly compared with the data from the focus groups and the publications. The emerging findings were critically examined throughout the process by the authors in order to check the relevance and validity of the findings. Finally, the functionality categories were compiled with descriptions and presented in Table 3. The design categories were sorted according to the seven principles of Universal Design [30] and presented in Table 4 .

**Table 3:** Focus group participants’ requirements of the functionalities of the SALIG device for persons with cognitive impairment

Icons	Requirements of the functionalities of the SALIG device
Contacts	Picture dialling. Email and SMS.
Calendar	Options:
(Shared)	Daily, weekly and monthly views. Schedule tasks and reminders (voice and text). Record own reminders. Schedule repeated tasks: Daily, Monday-Friday, Saturday-Sunday, Weekly, Monthly, Annually. Schedule important tasks that need to be confirmed. Reminders to confirm that prioritised tasks are completed. Confirmation of prioritised tasks. Check if prioritised tasks have been confirmed. SOs and FCs notified if a task has not been confirmed.
Monitoring	Options:
	Monitor status of equipment at home. Receive reminders if for example windows, door of refrigerator, front door are not closed or if tap or cooker are not turned off. SOs and FCs notified. Possible for SOs and FCs to turn off equipment remotely.
	SOs and FCs notified in the event of a fall.
	Reminders if meals are not taken at scheduled times. SOs and FCs notified if meals are not taken at scheduled times.
Pillbox	Options:
(Synchronised with electronic Pillbox)	SOs and FCs notified if the medication is not taken.
	Medication schedule.
	Information if medication should be taken with food.
	View next scheduled dose of medication.
	View if medication is taken.
	Purpose of taking medication.
	Information about the user’s medication allergies.
	Picture of medication for recognition.
Care plan	View current care plan and previous care plan.
Help me	Emergency call to 112.
	Support call to a preselected SO for non acute problems via picture dialling.
Settings	Mainly for SOs and FCs.
Personal:	Register name, role, email, telephone numbers to SOs and FCs.
Functions:	Options: Calendar, Monitoring, Communication, Medication, Care plan, Help me.
Look &feel:	Set language (Dutch, English, Spanish, Swedish), font size and colour style. Adjustable volume for video calls, voice reminders. Choose ringtone. Choose digital or analogue clock.
Help me:	Select a contact to support non acute problems. Register a telephone number as default and if no answer the system should automatically switch to another registered telephone number.
Contacts:	Edit name and role, telephone numbers, email, picture.
Calendar:	Options:
	Lock editing function for persons with cognitive impairment.
	Reminders: Choose 1, 2 or 3 voice reminders after 5, 10 and 15 min time interval as default.
	Jingle: Choose from list or upload own jingle.
	Display only current monthly view.
	Voice message when tapping a scheduled task.
	Reminders for charging the battery as default.
Other	Login with fingerprints or voice password.
	A shared “To-do list”.

**Table 4:** The focus group participants’ recommendations of the design of the SALIG device for persons with cognitive impairment presented according to the seven principles for universal design

The seven principles for universal design	The focus group participants recommendations of the design (*n* = 14)
1. Equitable use	The design should not stigmatise
	The design should be attractive to the users
	The design should be age-relevant
2. Flexibility in use	Flexible design
	Compatible with other types of technologies
3. Simple and intuitive use	Require minimal new learning
	Easy to use and understand
	Self-instructive
	Not require a chain of actions
	Step-by-step instructions should be easy to understand
	Give feedback on all actions
	Consistent navigation and design
	Do not use one button for two functions
	Arrows and drop lists should not be used
	Require minimal maintenance
4. Perceivable information	Display only necessary and relevant information
	Clear and simple text
	Text message displayed in the middle of the screen
	Colour alone should not carry information
	Similar language and concepts
	Large, easy to understand standardised graphical symbols
	Blinking, animated icons or graphics should not be used
	Headlines included in pictures
	Upper and lowercase letters
	Same font
	Italic font should not be used
	Possibility to choose large fonts
	Clear letter spacing between each character
	Shadow effect should not be used
	Light background with black text
	High level of contrast
	Buttons should be clear and large
	Colour shifting should not be used
	Patterned background should not be used
	Good sunlight readability
	Different modes for reminders (alarm signal, text or voice message)
	Different ways of being alerted (light, jingle
	Good sound quality
	Possibility to adjust volume
	Clear indication for low battery level
5. Tolerance for error	Provide warnings of hazards and errors
	Give visual or/and verbal feedback for each step that is performed
	Must be reliable and robust
	Give guidance questions that could be answered by yes/no buttons or voice commands
	Prevent mistakes
	Correct errors
6. Low physical effort	Comfortable to use
	Possibility to adjust the pressure sensitivity of the touch-screen
	Cause a minimum of fatigue and be easy to handle for people with decreased physical strength and inferior fine motor coordination
7. Size and space for approach and use	Have a cover that allows variations in hand and grip size

#### Phase 3. Development of a design concept

In phase 3, Human and Interaction designers in the project created a preliminary design concept in PowerPoint, based on the users’ requirements and needs. This was evaluated by our research group, which comprised participants with high clinical and scientific experience regarding cognitive impairment. One Webmaster with design experience was also included. The research group discussed several versions of the concept and gave feedback to the designers. It was an iterative process of feedback and refinement of the design concept.

#### Phase 4. Develop and evaluate a first mock-up

**Fig. 1:**
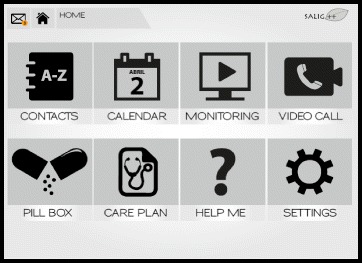
Image of the functionalities of the mock-up

Based on the design concept a first mock-up was developed. The following eight functionalities were included in the mock-up: Contacts, Calendar, Monitoring, Video Call, Pillbox, Care plan, Help me, and Settings (See Fig. 1).

The next step was to evaluate the mock-up. The purpose of the evaluation was to visualise the functionalities of the mock-up for the users in an early stage of the development and to examine how the design could be further developed and improved to meet the users’ needs and requirements. Despite the structured development process which follows the principles of inclusive design [24] the first mock-up was too abstract and rudimentary to be evaluated by persons with cognitive impairment and the evaluation therefore focused more on health care professionals. They had to be working with persons with cognitive impairment and have more than 2 years’ experience in this position. Thereafter, we invited persons with cognitive impairment and SOs to participate. Persons with cognitive impairment and SOs were recruited through a rehabilitation unit in Stockholm. Inclusion criteria for persons with cognitive impairment were that they had to be able answer open-ended questions, had a reasonable capacity to remember daily events and relate them to their cognitive problems and had a need for increased independence through use of AT. Exclusion criteria were other concurrent somatic or psychiatric disease influencing participation. SOs to persons with cognitive impairment had to be a spouse, relative or a close friend significantly familiar with the everyday life of a person with cognitive impairment. Potential participants were invited and informed by telephone and in writing. For a description of the participants’ characteristics see Table 5 and for their use of ICT see Table 6.

**Table 5:** Characteristics of the participants in the evaluation of the mock-up (*n* = 12)

Persons with cognitive impairment (*n* = 2)	
Male/female	1/1
Age, range	32, 36 years
Diagnosis	Stroke
Cognitive impairment	Memory deficits
Time since onset	7, 13 months
Compensatory strategies	Memos, mobile phone reminders
Health care professionals (*n* = 9)	
Occupational therapists (OTs)	5
Nurses	3
Assistive nurse	1
Age, median (range) SD, years	43 (23–63) 11.5
Professional experience, median (range) SD years	15 (2–23) 6.9
Significant other (*n* = 1)	
Relationship to person with stroke (PS)	Spouse
PS cognitive impairment	Neglect, attention deficits
PS time since onset	7 years
PS need of support	24 h assistance

**Table 6:** Mock-up evaluation participants’ use of ICT (*n* = 12)

ICT	Persons with cognitive impairment	Health care professionals	Significant other
	(*n* = 2)	(*n* = 9)	(*n* = 1)
Computer	2	9	1
Tablet	1	7	1
Smartphone	1	8	1
Smart TV	0	5	0

An interview guide with open questions was developed for the evaluation covering the design and the functionalities of the mock-up. The first author carried out the interviews and the second author took field notes during the evaluation sessions. The interviews were conducted either in the hospital or in the participants’ homes. All interviews were recorded and lasted between 45 and 60 min. Each interview started with a presentation of the purpose of the study and a short explanation of how the evaluation of the mock-up would be conducted. After that, the mock-up was demonstrated in a tablet (Ipad). It was not possible to show the “Pillbox”, “Care Plan” and “Help Me” functionalities since they had not yet been developed. The participants were interviewed about the relevance of the functionalities, what was missing, how useful the design was for persons with cognitive impairment and how the design could be improved. After each evaluation session the material was listened through and data that described important issues regarding the design and functionalities of the SALIG device was identified and transcribed. The researchers also reflected on the data and discussed issues that should be brought up in the next interviews. This meant that the interview guide was modified between the evaluation sessions according to the principles of grounded theory [29] in order to capture issues relating to the design and functions that had not previously been addressed. After 12 evaluation sessions the data was considered to be rich enough to shed light upon how to improve the design and functions of the mock-up and no further participants were recruited. The analysis of the collected data followed the same procedure as described in phase 2. The findings from the evaluation of the mock-up are presented in relation to the functionalities and the design of the mock-up.

## Results

### Functionalities of the mock-up

Overall, the persons with cognitive impairment, health care professionals and SO were satisfied with the functionalities of the mock-up. All of them agreed that the main benefit was that it was a single device with multiple functionalities. The OTs pointed out that most ATs on the market could only support one single need.

All participants appreciated the fact that the calendar could be shared with SOs and accessed from different locations. Some of the OTs stressed that even though the person with cognitive impairment could receive reminders for scheduled tasks there is a risk of forgetting, for example if the reminder occurs during an on-going task. In order to keep track of whether a task have been carried out the participants suggested that the person with cognitive impairment should confirm in the calendar that the task has been completed. Moreover, the participants thought that medication reminders are very important as persons with cognitive impairment might forget to take their medication. They also said that if, despite reminders, medication is not taken SOs and FCs should be informed via email or SMS. The nurses came up with suggestions of functionalities that could be included as optional in the functionality “Pillbox”. They suggested that it should be possible to check the next scheduled dose, to check if medication is taken, to view the medication schedule and a list of current medication including information and special instructions, such as to be taken with a meal or before bedtime as well as the purpose of taking the medication. Moreover, it could be important to have information about the user’s medication allergies, for example when contacting physicians and pharmacies. The nurses also suggested that pictures of the medication could be added for recognition.

The participants thought that a picture dialling function could make it easier to make calls and maintain social contacts with SOs. They suggested that a picture of the person dialling should be displayed on the screen to facilitate recognition. In order to further enhance usability the picture should be large and clear. The persons with cognitive impairment and SO thought that persons with cognitive impairment should have maximum ten contacts for video calls. Thus, alphabetical search or contact categorisation is not needed.

The participants were very positive to the functionality “Monitoring” that could be used to monitor the status of equipment in the home environment and send event reminders to the user if, for example, the coffee machine is not turned off or if windows or the front door are not closed. They thought that event reminders could be beneficial in order to prevent dangerous situations if, for example, the person with cognitive impairment has forgotten to turn off the tap or the cooker. Moreover, they said that it should be possible for SOs and FCs to turn off equipment in the home remotely. Another important request was to receive SMS or email if the person with cognitive impairment had fallen or forgotten to have a meal. A particularly important functionality was “Help me”. They suggested that picture dialling for emergencies (emergency telephone number) and a picture of SO for non acute situations could facilitate the use of the function.

Surprisingly, the functionality “Care plan” was considered less important. Only some of the nurses and OTs mentioned that it could be useful, but they did not further elaborate on this issue. However, they did point out that only one document should be displayed in “Previous care plans” since too many “previous care plans” could be confusing for persons with cognitive impairment.

Moreover, the persons with cognitive impairment and SO asked for a digital or analogue clock that should be displayed in all menus. To decrease the amount of information, a second hand should not be displayed. A shared to-do list was another requested function as some tasks could not be time scheduled.

### Design of the mock-up

The participants appreciated that the device was installed in a commercial product (tablet). They pointed out that the design of ATs is often unattractive and is not modern, which could stigmatise or make the user seem different in some way. Even though it might be difficult for persons with impaired fine motor skills to use a touch-screen, the OTs thought that it could be a useful feature.

The most important requirement of the design was that device should be easy-to-use. The OTs pointed out that the user interface should be intuitive, self-instructive and only communicate necessary information that is clear and easy to understand. For example, they thought that the user interface of the calendar was unclear and that it probably would be too complex for persons with cognitive impairment to use. The OTs recommended that unnecessary details and steps should be removed and that headings and texts should be clear and simple. Some of the OTs said that persons with cognitive impairment might have problems understanding how to use a drop-down list with a scrolling function. The OTs commented that even if standardised icons were used in the mock-up some of them could be difficult for persons with cognitive impairment to recognize. Some OTs recommended using standardised graphical symbols that are easy to understand for persons with cognitive impairment. Another important requirement was that the design should be flexible. The users’ individual needs and abilities should be taken into account as well as the changing needs in everyday life. The OTs also said that SOs or FCs would play an important role in setting up and personalise the device and in supporting the use and management. Furthermore, some of the OTs suggested that the device should have a pre-set standard menu which allows for setting adjustments as many options could be too difficult and time consuming.

The participants provided detailed information on how reminders could be designed to be easy-to-use for persons with cognitive impairment. For example, default recorded voice reminders should be short, pleasant and clear and it should be possible to record your own reminders. Timed text message reminders should be displayed on the screen until the last voice message reminder has been given. Furthermore, they suggested that colours could be used in the calendar to indicate if a task has been completed and confirmed. In order to increase readability, the OTs recommended that past time in the calendar could be displayed in a contrasting colour, that blank cells in the monthly view of the calendar could be greyed out and that the current date could be highlighted. Furthermore, they said that persons with cognitive impairment might have difficulties in remembering passwords and they should only have to login the first time they use the SALIG device. However, SOs and FCs should have to login each time.

## Discussion

The goal of the SALIG project was to develop an ICT-based device that could monitor and respond to the needs of the users’ with respect to support in maintaining an independent life despite their cognitive impairment. The findings from the mock-up evaluation showed that the main benefit of the mock-up was that it was a single device with multiple functionalities installed on a tablet and designed for persons with cognitive impairment. An additional benefit was that it could be used remotely by SOs and FCs. Moreover, the findings showed that persons with cognitive impairment prefer an all-in-one device, as today they often need several different ATs. This implies that the SALIG device might make obsolete the use of a large number of different devices such as alarm clocks, calendars, telephones, and, navigation tools. However, there could be a risk attached to being dependent on a single device if it fails or if the user forgets to charge it or loses it [2, 13].

The participants had overall a very positive attitude to the functionalities of the mock-up. The most important functionality was the shared”Calendar” that could be accessed by SOs and FCs. Earlier studies of electronic calendars such as “Google Calendar” have found that they could facilitate the planning of tasks, to ensure that tasks are entered correctly and that timed text message reminders are useful [2, 5, 31]. The SALIG device had several new functionalities compared to Google Calendar such as voice message, a function for the confirmation of important tasks and if the tasks are not confirmed an SMS will be sent to SOs or FCs. In line with previous studies [4, 32], the participants requested a function to confirm important activities since persons with cognitive impairment might have difficulties in recalling if activities had been carried out. There are few ATs on the market that incorporate such a function. A problem with existing ATs is that several steps are required to confirm an activity and therefore, too difficult for persons with cognitive impairment to learn how to use. Thus, a self-instructive and easy-to-use function for confirmation of completed activities is needed. Furthermore and in line with earlier research, the results indicated the need to unburden the SOs [33, 34]. The proposed design might be a step towards this goal.

Another important functionality was the Pillbox that could remind the person with cognitive impairment to take their medication if it has not been taken at prescheduled times. In addition, SOs could receive an SMS if the medication has not been taken in spite of reminders. It has been pointed out in the literature that a major problem for persons with cognitive impairment could be remembering to take their medication and SOs often have to remind them and monitor that medication is taken [14, 35]. Even if medication compliance is recognized as an important issue within health care, not many studies have examined medication compliance using new technologies [14, 36]. In order to make it easier for persons with cognitive impairment to keep track of their medication the participants recommended including several new useful functionalities in the Pillbox icon.

The findings also showed that there should be no stigma associated with the design of the device. The participants appreciated that the SALIG device was installed on a tablet designed to be used by all kinds of people. Previous studies have underscored that to be accepted, the design of devices for persons with cognitive impairment should be aesthetic and not look like AT or medical equipment [10]. In addition it is important that the device is consistent with the users’ life style [2]. One way of addressing this is to develop solutions that are based on technology that is commonly used and accepted by today’s society [9]. However, earlier studies have shown that if persons with cognitive impairment are to be able to use a commercial technologies such as a smartphone independently as a compensatory device, a structured training programme might be needed [5, 37]. Furthermore, persons with cognitive impairment often need support from SOs in maintaining a smartphone [37]. It is well-known that the responsibility for supporting persons with cognitive impairment often lies with the SOs [38]. Caring for a person who has cognitive impairment can be a demanding task and can increase stress and decrease well-being for SOs [34]. Research has mostly focused on developing technology solutions to support persons with cognitive impairment to increase independence in everyday life. Thus, the SALIG device should also be developed to unburden SOs.

Although, the mock-up was not fully developed the persons with participants provided important suggestions concerning how the design could be improved to meet the users’ needs and requirements in an early phase of the development process. There were a number of suggestions on how the design should be further developed to be easy and sufficiently self-instructive to allow for intuitive use. This is in line with other studies that has found that the user interface have to be easy-to-use and understand in order to increase the possibility for persons with cognitive impairment to us it independently [7, 13, 19, 39]. It has been highlighted in the literature that being able to use a device independently is important for long-term use [39, 40]. Furthermore, Boman and colleagues [10] have pointed out that it is important that the design is flexible in relation to the user’s individual and changing needs. Considering this, it is important that the SALIG device is easy to customize. Earlier research has showed that SOs and FCs play an important role in adapting the device and support use and management [12]. If there are too many options it might become difficult for SOs to identify which functionalities that are needed and to adjust them according to the user’s current situation. One suggestion would be to have three different packages in order to facilitate customisation.

It has been highlighted in earlier studies that developers need to have an understanding of the end-users’ needs and requirements in order to develop useful devices [41]. Otherwise, there is a risk that the development process of new devices is technology-driven, i.e., technologies are developed based on what is technological possible [42, 43]. Poorly designed devices can be a challenge for end-users or at worst be left unused. In this study, Universal Design [30] were used to categorise and present the design requirements. Several of the design requirements fitted into Principle 3: Simple and intuitive use and Principle 4: Perceivable information. This implies that the design requirements in these principles should be specifically in focus when developing devices for persons with cognitive impairment.

A limitation in this study was that the sample size was rather small and consisted of a convenience sample. Though the sample was rather small, the data was considered to be rich enough to cover the study issues. However, there is a risk that the respondents did not cover the variety of needs of the target group and the generalisation of findings should be made with caution. The needs and requirements of the target group had been documented in the form of a user requirement specification. There have been some challenges in translating these requirements into the design and several functionalities in the mock-up were not developed yet. Therefore, it was not possible to show the participants what the design will look like with respect to all functions. For that reason we included only two persons with cognitive impairment in the mock-up evaluation. It has been pointed out in the literature that persons with cognitive impairment should not be involved in an early phase of the development of new products since exposure to poorly functioning technology might cause confusion and frustration [44]. Moreover, persons with cognitive impairment could have difficulties to imagine things they cannot see and to get an overall idea of how a device is intended to be used [1]. They might also have difficulties to articulate their perceptions, reflect and come up with new ideas and to answer open questions [45]. Considering this health care professionals working with persons with cognitive impairment were included in the mock-up evaluation as it is recommendations in the literature [46]. Furthermore, it is important to involve end-users throughout the development process. Otherwise, there is a risk that the development process of new devices is technology-driven, i.e., technologies are developed based on what is technological possible [42, 43].

## Conclusions

In summary, the SALIG device has potential to facilitate everyday life for persons with cognitive impairment and unburden SOs and it could also be a useful tool for formal caregivers. The main benefit of the SALIG device was that multiple functionalities were included in a single device. An additional benefit was that it could be used remotely by SOs and FCs. The evaluation of the mock-up in an early stage of the development process provided comprehensive and valuable information on how the functionalities and the design of the SALIG device could be further developed to be more useful for persons with cognitive impairment, their SOs and FCs. The findings in this study should not only guide the future development of this device, they might also provide guidance in the development of various types of technology for the target population and people with diverse disabilities.
